# Detection of Acipenser European Iridovirus (AcIV-E) in Sturgeon Farms in Northern Italy between 2021–2023

**DOI:** 10.3390/v16030465

**Published:** 2024-03-18

**Authors:** Fabio Bondavalli, Dáša Schleicherová, Paolo Pastorino, Davide Mugetti, Claudio Pedron, Marino Prearo

**Affiliations:** 1 Istituto Zooprofilattico Sperimentale del Piemonte, Liguria e Valle D’Aosta, 10154 Torino, Italy; fabio.bondavalli@izsto.it (F.B.); dasa.schleicherova@izsto.it (D.S.); marino.prearo@izsto.it (M.P.); mugetti@aslvco.it (D.M.); 2 Department of Life Sciences and Systems Biology (DBIOS), University of Turin, Via Accademia Albertina 13, 10123 Turin, Italy; 3 ASL VCO, Department of Public Health, Omegna Health District, Via G. Mazzini, 96, 28887 Omegna, Italy; 4 DVM, 20090 Settala, Italy; claudio.pedron@alice.it

**Keywords:** *Acipenser* spp., AcIV-E, *Huso huso*, sturgeon farming

## Abstract

Sturgeon farming is rapidly expanding in Europe, where Italy ranks first in farmed caviar production. A major threat to sturgeon health in captivity is infection with Acipenser European Iridovirus (AcIV-E), a viral disease definitively identified in 2016. Here we present data on the occurrence of AcIV-E in 482 sturgeons (age ≤ 12 months, species of the genus *Acipenser* and the species *Huso huso*) collected from sturgeon farms in northern Italy between January 2021 and December 2023. The health status of each specimen was determined by necroscopy and virological assay. Virological analysis was performed on gill samples and real-time PCR specific to the MCP gene of the iridovirus viral capsid. Molecular analysis revealed positivity to the virus in 204 samples (42.68% of the total), while anatomopathological examination of nearly all fish with positive real-time PCR disclosed swollen abdomen, hepatic steatosis, splenomegaly, and increased gill volume. Two challenges to timely diagnosis are the absence of pathognomonic symptoms and the inability to isolate the virus on cell monolayers. Continuous and widespread health monitoring is therefore crucial for disease management and to effectively control spread of the virus.

## 1. Introduction

Sturgeon farming is expanding in Europe, where Italy ranks first in caviar production, with 25 tons in 2013 alone [1,2] from five species: Siberian sturgeon (*Acipenser baerii*) (30.9%), Russian sturgeon (*A. gueldenstaedtii*) (20.4%), the hybrid *Huso dauricus* × *A. schrenckii* (13.1%), white sturgeon (*A. transmontanus*) (12.1%), and sterlet sturgeon (*A. ruthenus*) (5.2%) [2].

The wild sturgeon population has declined in both the number of species and individual abundance due to overfishing [3,4] and to the introduction of non-native species [5]. While sturgeon aquaculture farming has bolstered caviar and meat production [6,7,8] it also raises concerns about breeding methods. Being anadromous, sturgeons are generally more resilient than other species to certain diseases in their natural habitat; however, infectious disease can seriously impede the current expansion of sturgeon farming.

Bacterial and parasitic diseases [9,10] are generally manageable with therapeutic treatment, whereas viral infection poses a greater challenge due to the lack of effective therapies [11]. Although fragmented, available genomic data allow the classification of these viruses as nucleocytoplasmic large DNA viruses (NCLDVs) [12,13]. Sturgeon NCLDVs make up a large group of viral agents: White sturgeon iridovirus (WSIV), Shortnose sturgeon virus (SNSV), Missouri River sturgeon iridovirus (MRSIV), British Columbia white sturgeon virus (BCWSV), and Namao virus (NV). While NCLDVs are not officially recognized by the International Committee on Taxonomy of Viruses (ICTV) [12,13], they show close similarity with Mimiviridae, like Acipenser iridovirus-European (AcIV-E) for example. The order also includes viruses of the Mimiviridae family, which are closely related to pathogenic sturgeon viruses [13]. These taxonomic data, together with two different variants of AcIV-E, require a revision of the current nomenclature [13]. However, while AcIV-E seems to be closely related to North American sturgeon iridoviruses, particularly WSIV and NV, there is limited data regarding the presence of NV in Europe [12,13]. Furthermore, NV has not been identified in Italy, so there is no bibliography available to support its presence.

Russian sturgeon seems to be the species most susceptible to AcIV-E [12,14], though mortality has also been reported in other species, including *A. naccarii*, *A. baerii*, *A. stellatus*, *A. ruthenus*, and *H. huso* [12,15,16]. Clinical signs include anorexia, lethargy, and irregular swimming [12], while cutaneous ulcers, gill hypermucosity, and other visual signs are not considered diagnostic. Furthermore, young individuals appear more susceptible to infection than adults. Sturgeons are also susceptible to diseases caused by opportunistic pathogens or environmental bacteria: *Aeromonas* spp. (*A. hydrophila*, *A. sobria*, *A. veronii*) [10,17,18,19,20,21,22], *Acinetobacter* spp., *Hafnia alvei* [10], *Yersinia ruckeri* [10,23,24,25], *Pseudomonas* spp. [10,21,26], *Citrobacter freundii* [21,27,28], *Plesiomonas shigelloides* [10,28,29], and *Flavobacterium* spp. [21,30,31].

The primary diagnostic approach to identifying AcIV-E involves real-time PCR [15]. Though histological analysis can also be conducted, conventional virological testing using cell lines (BF-2, CCP, EPC, GF, SSN-1, E11, WSSK) is ineffective as the virus does not exhibit growth and subsequent cytopathic effects [15].

Here we present data on the occurrence of AcIV-E infection in sturgeon farmed in the Po Valley, northern Italy, over a three-year period (2021–2023). Virological analysis was performed following the protocol for molecular methods as described by Bigarré et al. [15].

## 2. Materials and Methods

### 2.1. Sampling

The sample consisted of a total of 482 specimens exhibiting diverse health problems and experiencing tank mortality. Specimens were collected from eight farms between January 2021 and December 2023. The sample comprised different species of the genus *Acipenser* spp. and *Huso huso* (age ≤ 12 months): *A. gueldenstaedtii* (n = 257), *A. stellatus* (n = 56), *A. baerii* (n = 50), *A. naccarii* (n = 47), *H. huso* (n = 33), *A. transmontanus* (n = 22), and *A. ruthenus* (n = 17) (Table 1 and Table 2).

Samples were placed individually in a plastic bag and sent by breeders or breeding technicians directly to our laboratory, refrigerated in a controlled environment generated by portable refrigerators cooled by eutectic plates (+4 °C). Moribund subjects had been extracted from the breeding tanks and were euthanized with a lethal dose of tricaine methanesulphonate (MS-222; 150 mg/L; Sigma-Aldrich, Milan; Italy), according to current regulations.

### 2.2. Anatomopathological Examination

Samples arrived from the farm to our lab within a maximum of 5 h under refrigerated conditions. Anatomopathological examination identified and documented lesions, both external and those within the visceral cavity.

### 2.3. DNA Extraction and Real-Time-PCR

DNA extraction from the gills was performed using a genomic DNA isolation kit (Extractme Genomic DNA, Blirt, Poland) following the protocol for fresh or frozen solid tissue. For real-time PCR (qPCR) targeting the MCP gene, a fragment of the major capsid protein of AcIV-E was used as described by Bigarré et al. [15] and using a PlatinumTM Quantitative PCR SuperMix, UDG kit (Invitrogen, ThermoFisher Scientific, Waltham, MA, USA). The reactions were conducted in 25-µL volumes, using 10 µM of each primer (oPVP346: 5′-TCAAAGTCTGGGACCTCTA-3′ and oPVP347: 5′-AGAGATGTTCAACTGGATGT-3′), the probe tqPVP20: 5′-FAM-TTGTGAATCATATCGCCAGTCAT-BHQ1-3′, and 5 µL of the extracted DNA. Nuclease-free water served as the negative control, while a plasmid with the AcIV-E MCP gene sequence was the positive control for each PCR. Reactions were performed on a StepOnePlus™ real-time PCR System (Applied Biosystems, Foster City, CA, USA). The MCP gene was amplified as follows: initial denaturation at 95 °C for 15 min, followed by 40 cycles of denaturation at 94 °C for 15 s, and annealing at 60 °C for 60 s [15].

## 3. Results

### 3.1. Virological Analysis

A total of 204 sturgeons tested positive for AcIV-E (Table 1).

**Table 1:** Real-time PCR results by sturgeon species.

Species	No. of Subjects	Positive (%)	Negative (%)
*Acipenser gueldenstaedtii*	257	164 (63.81)	93 (36.19)
*Acipenser baerii*	50	6 (12.00)	44 (88.00)
*Acipenser ruthenus*	17	2 (11.76)	15 (88.24)
*Acipenser transmontanus*	22	0	22 (100)
*Acipenser naccarii*	47	19 (40.43)	28 (59.57)
*Acipenser stellatus*	56	13 (23.21)	43 (76.79)
*Huso huso*	33	0	33 (100)

The farmers consistently reported noticeable symptoms of evident anorexia and swimming ataxia characterized by superficial and uncoordinated movements. Sick fish often exhibited passive transport by the current towards the tank grids and displayed swimming ataxia when stimulated.

Table 2 presents the positivity rate by species and year. *A. gueldenstaedtii* was most often affected, likely because of its heightened susceptibility to tank mortality. The positivity rate increased from over 61% in 2022 to over 72% in 2023. Although the sample base varies somewhat, the average positivity rate was 63.81%.

**Table 2:** Positivity rate by year and species.

Species	Year	Total	Positive (%)
*Acipenser gueldenstaedtii*	2021	136	86 (63.24)
	2022	85	52 (61.18)
	2023	36	26 (72.22)
*Acipenser baerii*	2021	0	0
	2022	20	4 (20.00)
	2023	30	2 (6.67)
*Acipenser ruthenus*	2021	0	0
	2022	10	2 (20.00)
	2023	7	0
*Acipenser transmontanus*	2021	2	0
	2022	10	0
	2023	10	0
*Acipenser naccarii*	2021	0	0
	2022	36	13 (36.11)
	2023	11	6 (54.55)
*Acipenser stellatus*	2021	10	2 (20.00)
	2022	21	10 (47.62)
	2023	25	1 (4.00)
*Huso huso*	2021	10	0
	2022	18	0
	2023	5	0

Another species subject to virus-induced mortality was *A. naccarii*, with a positivity rate of just over 36% in 2022 and over 54% in 2023 (average, 40.43%), followed by *A. stellatus*, with an average mortality rate of 23.21% (4% in 2023 and 47% in 2022). Differently, the positivity rate for *A. baerii* and *A. ruthenus* was <15%, except for peaks of 20% for both species in 2022. The average mortality rate for *A. stellatus* was 23.21% (4% in 2023 and over 47% in 2022). In contrast, *A. transmontanus* and *H. huso* tested negative for the virus across all 3 years of sampling, probably because of the relatively small sample size.

### 3.2. Anatomopathological Examination

**Figure 1:**
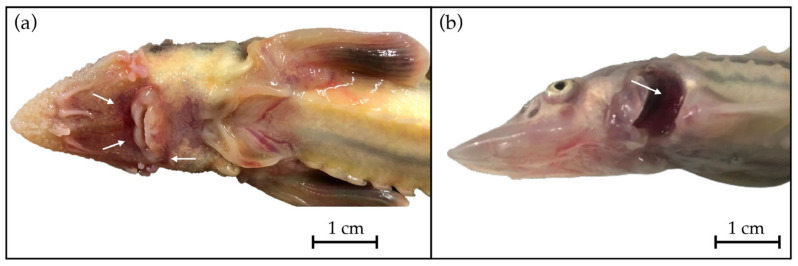
Anatomopathological examination of a Russian sturgeon (*Acipenser gueldenstaedtii*) revealed (**a**) redness around the mouth and (**b**) increased gill volume.

Necropsy revealed a tense and swollen abdomen in many subjects, accompanied by skin lesions, redness at the base of the fins and in the ventral part of the head (Figure 1a), and weight loss in the fish with severe symptoms.

Upon opening and viewing the opercular cavity, enlargement of the gill lamellar tips was evident (Figure 1b), often with a hemorrhaging of the gill epithelium, splenomegaly in the visceral cavity, hepatic steatosis in paucisymptomatic subjects coming from batches with lower mortality in which anorexia was not yet particularly evident. Food was often found in the gastrointestinal tract in less severe cases, whereas the stomach and the intestines were empty in those with obvious symptoms and very high mortality, while the liver appeared normal or marbled.

## 4. Discussion

The data from the 3-year study period (2021–2023) indicate AcIV-E infection as a primary health concern for sturgeon farming tin Italy. Approximately 42% (204/482) of specimens tested positive for AcIV-E. The variation in infection rate by sampling year and species suggests the dynamic nature of viral pathology.

Virological analysis showed that *A. gueldenstaedtii* was more often affected (164/257, 63.8%). This finding is shared by previous studies [12]. High positivity for the virus was also noted for *A. naccarii* (19/47, 40.4%). An accurate diagnosis of AcIV-E is crucial in this species given its status as a native species and its partial management for release into open waters during reintroduction initiatives [32,33,34,35]. Introducing infected or potentially virus-carrying subjects poses a substantial threat to both natural populations and reintroduction programs.

Lower positivity rates were observed for *A. stellatus*, *A. baerii*, and *A. ruthenus* (range: 23.2–11.7%). It remains unclear whether these species inherently possess greater resistance to the virus or if the rates were influenced by random factors in farming and sampling.

Across the 3-year study period we noted higher positivity rates for the winter than for the spring–summer months. In addition, morbidity and mortality were higher in juveniles under 6 months of age compared with the lower mortality rate in batches of subjects older than 6 months, as reported elsewhere [12,16].

The disease is endemic to the study area. We observed a shift in susceptibility to the virus also in larger fish, in which mortality was very low and symptoms were less specific. Indeed, age and size did not appear to be a determinant factor in the onset of viral infection. This observation is shared by previous studies that reported infection in both juvenile and adult individuals of the species *A. gueldenstaedtii, A. baerii*, and *A. naccarii* [1,15,36].

Diagnosing AcIV-E poses several challenges. One obstacle is the absence of characteristic alterations on anatomopathological examination, which precludes the identification of pathognomonic signs for definitive diagnosis. It has been suggested that the virus may exhibit greater tropism towards epithelial cells, leading to noticeable alterations in the gills [37]. While this may offer diagnostic clinical signs, gill lesions are not consistently apparent. Another potential indicator is the difference in size between healthy and infected individuals [12], yet this too is not conclusive for the presence of disease.

A greater challenge lies in the inherent difficulty of isolating the virus on a cellular monolayer, rendering molecular diagnosis the sole viable option [15,37]. The inability to perform virological examination of virus growth on a cellular monolayer and its subsequent identification through a cytopathic effect means that the virus cannot be isolated and cultivated in sufficient quantities, a crucial objective for studying the pathogen’s effect on tissues.

Moreover, obtaining the virus in cell culture holds paramount importance for understanding its evolutionary path and enabling the development of diagnostic serological tests to assess the in vivo immune status of affected and surviving subjects. Equally crucial are the identification of potential vaccine targets and exploration of prophylactic measures. The lack of data regarding the immune status of fish that have survived infection, coupled with an incomplete understanding of the pathogenesis of the viral agent, impedes global comprehension of the disease.

A third challenge involves assessing the true homology among viral strains affecting distinct species. This entails verifying whether the difference in positivity rates is the result of species predisposition to the virus or rather results from different viral variants. Additionally, exploring the degree of homology between viral strains residing in diverse river basins could reveal much needed clues to their identification; however, such studies are presently unattainable due to the inability to isolate the virus.

A limitation of the present study is the absence of statistically significant data for the true prevalence of AcIV-E in sturgeon farms in Italy. The sampling does not provide a comprehensive cross-section of the overall health scenario. Since all subjects came from batches with noted health issues, our study does not present data on the actual prevalence of AcIV-E.

Moreover, the absence of uniform sampling units across different fish species precludes a precise and concrete estimate of the sensitivity of farmed species to the virus. The challenges to executing such a study are the high cost of the subjects and the regulatory complexities associated with the Convention on International Trade of Endangered Species (CITES) regulations governing subjects raised on farms.

Ultimately, AcIV-E-induced mortality constitutes a substantial economic threat to a sector of Italian aquaculture. The presence of the virus in farm wastewater is also an ecological risk for wild sturgeon populations already severely impacted by years of overfishing. Currently, these populations are at their lowest levels in species abundance and individual numbers [16,38]. All sturgeon species are listed on the IUCN Red List, with 16 considered in danger of extinction, including those native to Italy. Given that sturgeons are primarily cultivated in aquaculture for caviar production and that the economic viability of a sturgeon farm relies on a meat market as well, all health issues impact farm profitability.

Considering the lengthy production cycle required to obtain the primary product (8–10 years for sexual maturity) and the fact that approximately 20 tons of sturgeon meat are produced for every ton of caviar, it is evident that any health problem exerts a considerable financial burden. Over the past two decades, sturgeon production in aquaculture has experienced rapid growth, predominantly driven by Chinese production, which accounts for around 84% of global output. Italy ranks first among European Union member states in production (nearly €7 million in 2020 alone), followed by Poland and Bulgaria [39].

In the context of global health, all sturgeon species face imminent threats, with some species classified as extinct in the wild. Sturgeon survival in the river and lake basins of the Northern Hemisphere where these species are indigenous is threatened by human activities. Intensive fishing and ecological fragmentation contribute largely to their depletion in the wild. Intensive fishing, driven by economic profit from the high value of caviar, is a major factor. Despite strict regulations in the trade and the growing production of caviar from farmed sources, poaching persists in some regions, exacerbating the numerical decline of natural sturgeon populations and resulting in genetic impoverishment.

The second major threat to sturgeon populations arises from environmental interventions that diminish or eliminate connectivity between ecosystems. Since most sturgeon species are anadromous, the construction of dams and barriers complicates their migration, fundamentally altering the ecological characteristics of rivers and impeding reproduction.

An additional concern linked to ecosystems is the widespread introduction of alien species, which threatens sturgeon survival in certain regions of the world. The spread of a viral disease in combination with environmental threats creates a scenario in which the management of these species in their natural habitats becomes increasingly difficult. The characteristics, pathogenicity, and extent of disease are not yet fully understood, adding to the complexity of the challenges of preserving sturgeon populations.

## 5. Conclusions

The absence of pathognomonic symptoms and the inability to isolate the virus on a cell monolayer pose major challenges to achieving timely diagnosis. To effectively control the spread of the virus, it is imperative to establish continuous health monitoring in collaboration with all stakeholders, including breeders, research centers, and management agencies. Additionally, monitoring and certifying the health of sturgeons released in aquaculture farms play a pivotal role in disease management.

Finally, maintaining ongoing surveillance of sturgeon populations can contribute to preventing harm to wild populations in the vicinity of farms. Monitoring efforts should be extended to wild populations and non-invasive diagnostic methods employed to ensure population health and optimal conservation.

## Data Availability

Data are contained within the article.
